# Supplementary material for: Epstein–Barr virus-based plasmid enables inheritable transgene expression in mouse cerebral cortex
Source: PLoS One. 2021 Sep 30;16(9):e0258026. doi: 10.1371/journal.pone.0258026 (PMC8483300; doi:10.1371/journal.pone.0258026)

S1 Fig. Persistent transgene expression in all known progeny of NPCs by IUE of the EB-oriP plasmid in adult mice.

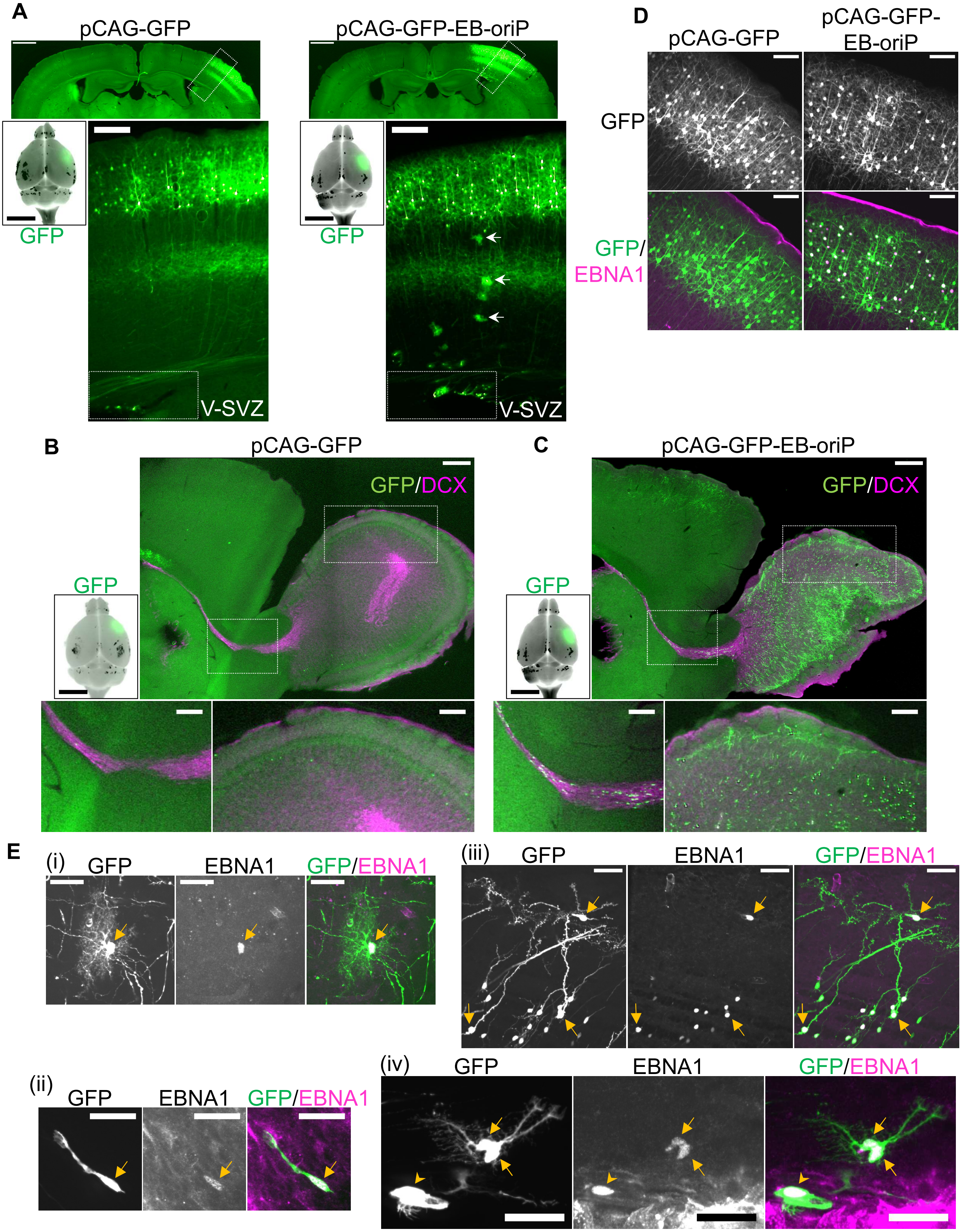

Supplement: S1 Fig — (A) GFP fluorescence images of the brains transfected at E15 with pCAG-GFP and pCAG-GFP-EB-oriP and fixed at P56. GFP fluorescent images of coronal sections (upper). Magnified images of the boxed regions in upper panels (lower right). Merged images of GFP fluorescence and bright-field image of transfected brains (lower left) used for sections shown in the upper and lower right. Scale bars: 5 mm. Scale bars: 1 mm (upper), 5 mm (lower left), 200 μm (lower right). (B and C) Sagittal sections of the brains transfected with pCAG-GFP (B) and pCAG-GFP-EB-oriP (C) and fixed at P56 were immunostained for DCX. Merged images of GFP fluorescence and bright-field image of the brains used for sections are shown in top left panels. The boxed regions in RMS (left) and OB (right) were magnified and are shown in bottom panels. Scale bars: 5 mm in top left panel, 500 μm in top right panel, 200 μm in bottom panels. (D) Representative magnified images of GFP-labeled neurons in superficial region of the brains transfected with pCAG-GFP and pCAG-GFP-EB-oriP, fixed at P56, and immunostained for EBNA1. Scale bars: 100 μm. (E) Representative GFP-labeled cells appeared to be an astrocyte (i), a migrating neuroblast in RMS (ii), neurons in OB (iii), and an ependymal cell and astrocytes in V-SVZ (iv) in the section of pCAG-GFP-EB-oriP-transfected brain fixed at P56 and immunostained for EBNA1. Scale bars: 25 μm in (i), (ii), (iv), 50 μm in (iii). (PDF) [file pone.0258026.s001.pdf]
