# Supplementary material for: Epstein–Barr virus-based plasmid enables inheritable transgene expression in mouse cerebral cortex
Source: PLoS One. 2021 Sep 30;16(9):e0258026. doi: 10.1371/journal.pone.0258026 (PMC8483300; doi:10.1371/journal.pone.0258026)
Supplement: S2 Fig — (A) Schematic illustration of plasmids encoding either EBNA1 (pCAG-GFP-EB) or oriP (pCAG-GFP-oriP), in addition to GFP. In utero electroporation into the lateral ventricle was performed at E15 and the brains were analyzed at P21 and P56. (B and C) (top) GFP fluorescence images of coronal sections from transfected brains at P21 (B) and P56 (C). (middle) Magnified images of the boxed regions in top panels. (bottom) Merged images of GFP fluorescence and bright-field image of transfected brains used for the sections shown above. Scale bars: 1 mm in top panels, 200 μm in middle panels, 5 mm in bottom panels. (D) Representative magnified images of GFP-labeled neurons in superficial region of the brains transfected with pCAG-GFP-EB (left) and pCAG-GFP-oriP (right) at P56 and immunostained for EBNA1. Scale bars: 100 μm. (E and F) Sagittal sections of the brains at P21 (E) and P56 (F) transfected with pCAG-GFP-EB (left, respectively) and pCAG-GFP-oriP (right, respectively) were immunostained for DCX. Merged images of GFP fluorescence and bright-field image of the brains used for sections are shown in upper left panels. The boxed regions in RMS (left) and OB (right) were magnified and are shown in lower panels. Scale bars: 5 mm in upper left panel, 500 μm in upper right panel, 200 μm in lower panels. (PDF) [file pone.0258026.s002.pdf]

S2 Fig. Sequences encoding both EBNA1 and oriP in the plasmid for IUE are required for transgene expression in postnatal progeny of NPCs

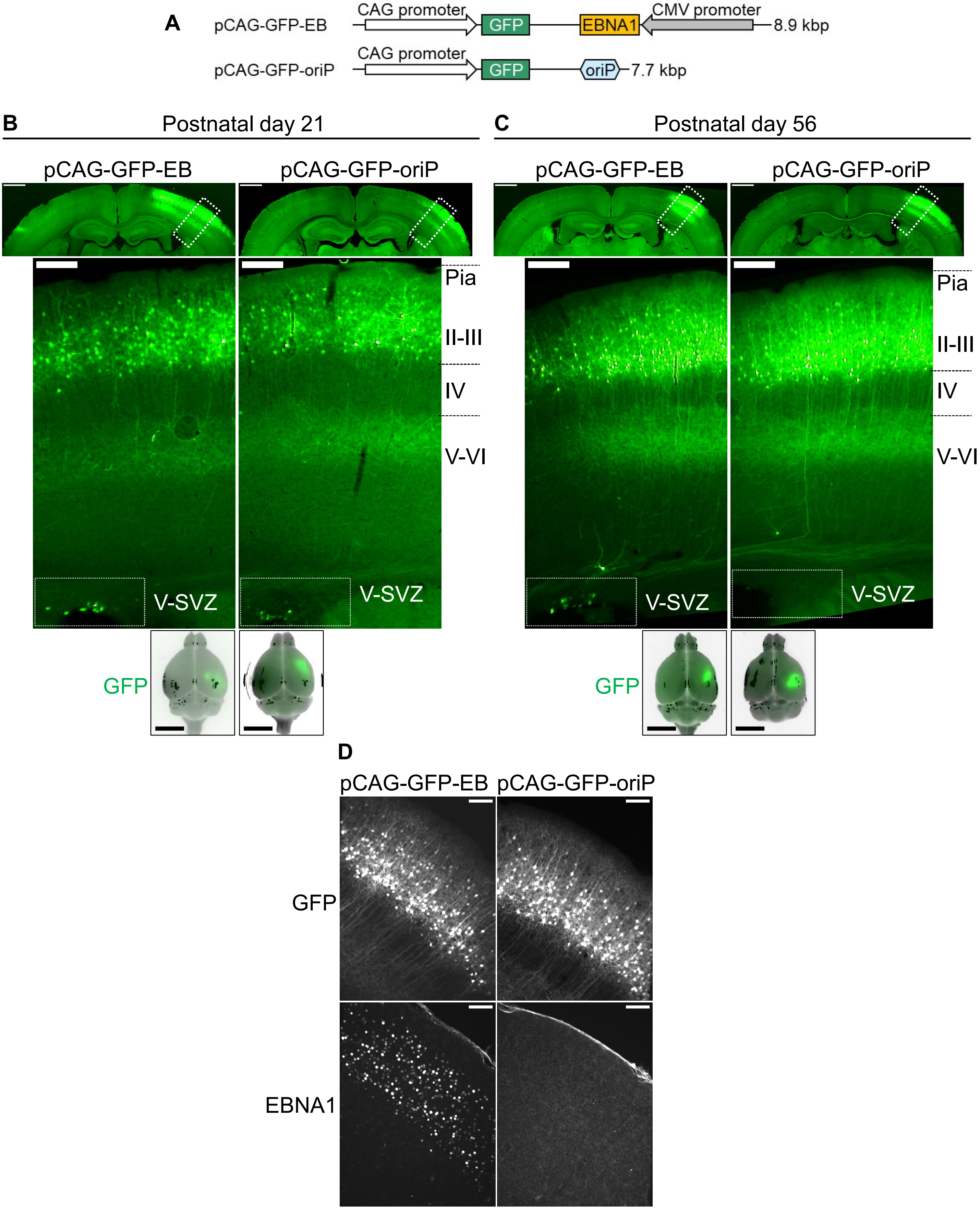

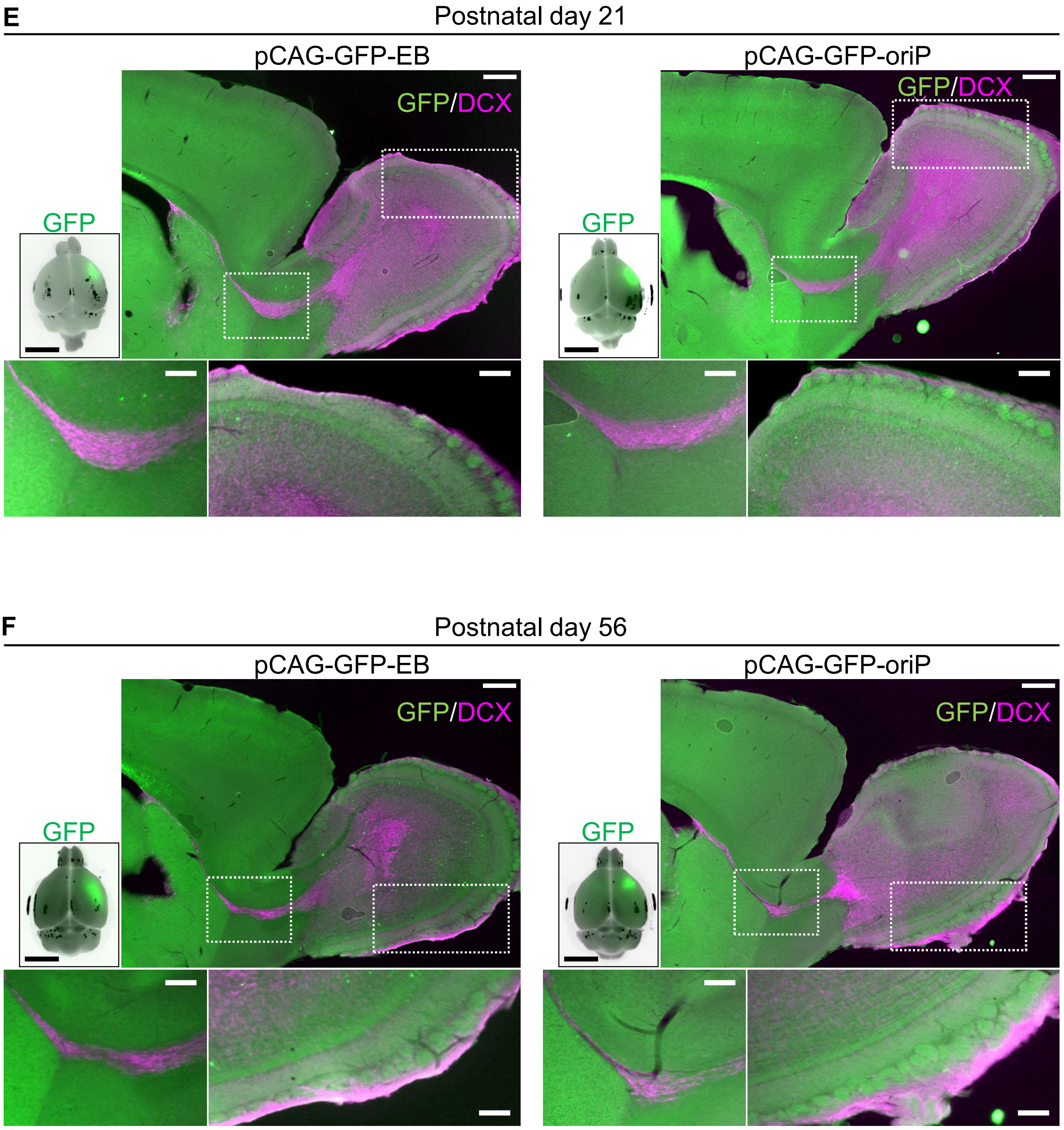
E
Postnatal day 21
pCAG-GFP-EB
pCAG-GFP-oriP
GFP/DCX
GFP
GFP/DCX
GFP
F
Postnatal day 56
pCAG-GFP-EB
pCAG-GFP-oriP
GFP/DCX
GFP
GFP/DCX
GFP
