# Supplementary material for: Epstein–Barr virus-based plasmid enables inheritable transgene expression in mouse cerebral cortex
Source: PLoS One. 2021 Sep 30;16(9):e0258026. doi: 10.1371/journal.pone.0258026 (PMC8483300; doi:10.1371/journal.pone.0258026)

S3 Fig. Transgene expression in not only neurons but also other postnatal progeny of NPCs by IUE of the EB-oriP plasmid in young adult C57BL6J mice.

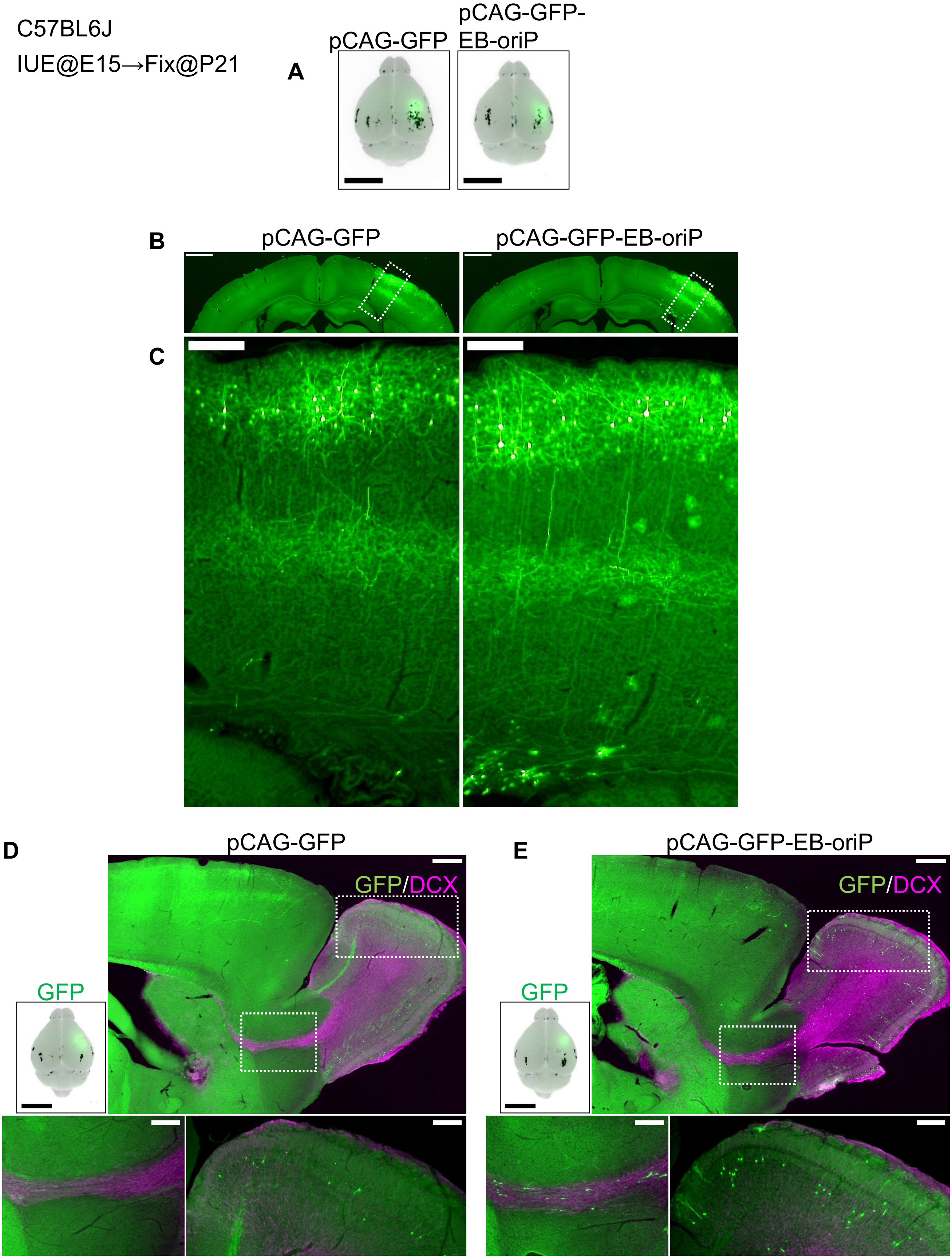

Supplement: S3 Fig — (A) Merged images of GFP fluorescence and bright-field image of the brains transfected with pCAG-GFP (left) and pCAG-GFP-EB-oriP (right). In utero electroporation into the lateral ventricle of C57BL6J mice was performed at E15 and observed at P21. Scale bars: 5 mm. (B) GFP fluorescence images of coronal sections from transfected brains shown in (A). Scale bars: 1 mm. (C) Magnified images of the boxed regions in (B). Arrows indicate GFP-labeled cells that morphologically appeared to be astrocytes. Scale bars: 200 μm. (D and E) Sagittal sections of the brains transfected with pCAG-GFP (E) and pCAG-GFP-EB-oriP (F) were immunostained for DCX. Merged images of GFP fluorescence and bright-field image of the brains used for sections are shown in upper left panels. The boxed regions in RMS (left) and OB (left) were magnified and are shown in lower panels. Scale bars: 5 mm in upper left panel, 500 μm in upper right panel, 200 μm in lower panels. (PDF) [file pone.0258026.s003.pdf]
