# Supplementary material for: Epstein–Barr virus-based plasmid enables inheritable transgene expression in mouse cerebral cortex
Source: PLoS One. 2021 Sep 30;16(9):e0258026. doi: 10.1371/journal.pone.0258026 (PMC8483300; doi:10.1371/journal.pone.0258026)

S4 Fig. Human β-interferon S/MAR sequence in the plasmid for IUE has little effect on persistent and inheritable transgene expression in postnatal progeny of NPCs in ICR mice.

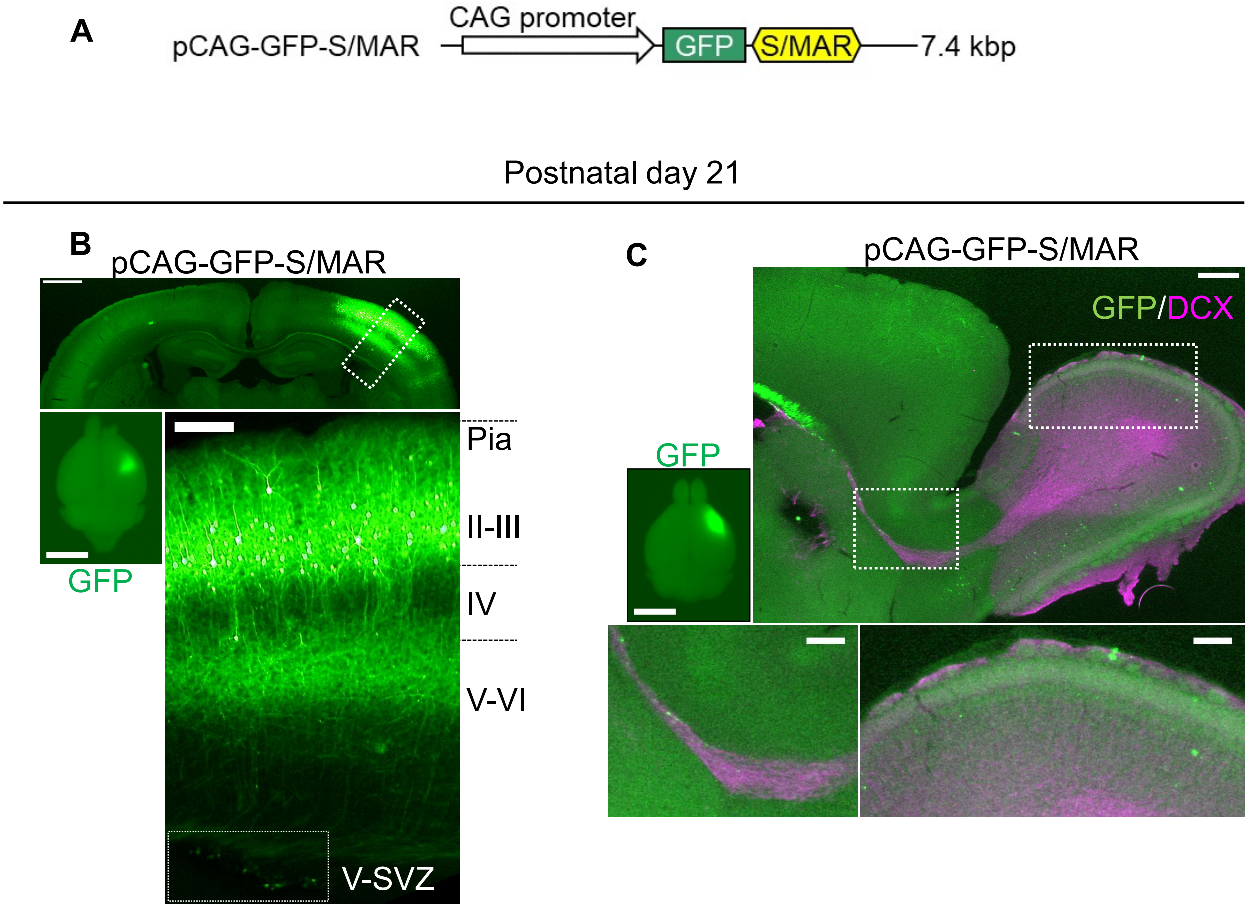

Supplement: S4 Fig — (A) Schematic illustration of the plasmid encoding human β-interferon S/MAR sequence, in addition to GFP. (B) A GFP fluorescence image of the brain transfected at E15 with pCAG-GFP-S/MAR and fixed at P21. A GFP fluorescent image of the coronal section (upper). A magnified image of the boxed region in upper panel (lower right). A GFP fluorescence image of the transfected brain (lower left) used for sections shown in the upper and lower right. Scale bars: 5 mm. Scale bars: 1 mm (upper), 5 mm (lower left), 200 μm (lower right). (C) A sagittal section of the brain at P21 transfected with pCAG-GFP-S/MAR was immunostained for DCX. A GFP fluorescence image of the brain used for sections are shown in the upper left panel. The boxed region in RMS (left) and OB (right) were magnified and are shown in lower panels. Scale bars: 5 mm in upper left panel, 500 μm in upper right panel, 200 μm in lower panels. (PDF) [file pone.0258026.s004.pdf]
