# Supplementary figures and images for: Epstein–Barr virus-based plasmid enables inheritable transgene expression in mouse cerebral cortex
Source: PLoS One. 2021 Sep 30;16(9):e0258026. doi: 10.1371/journal.pone.0258026 (PMC8483300; doi:10.1371/journal.pone.0258026)

S1\_raw\_images

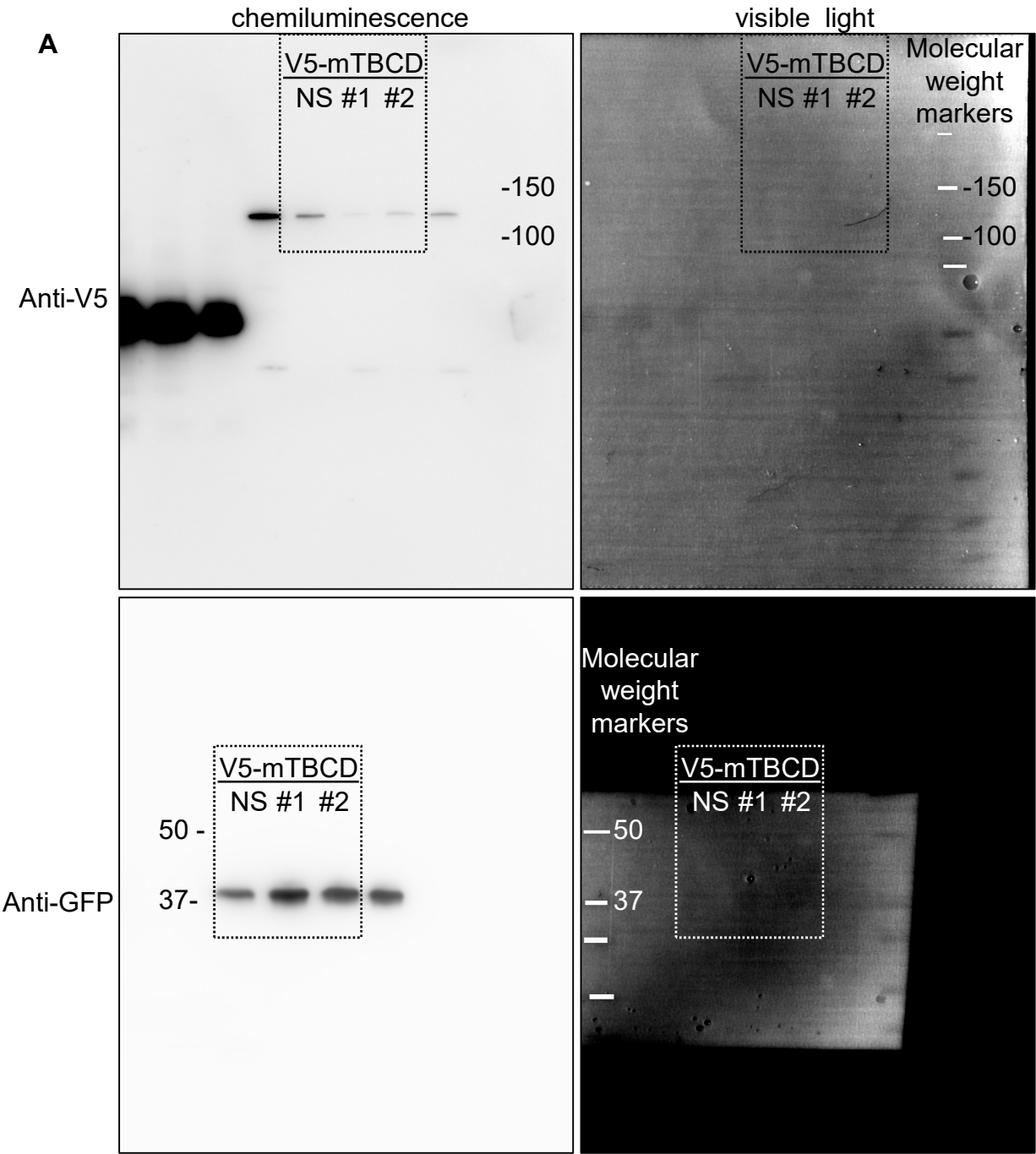

Supplement: S1 Raw images — (PDF) [file pone.0258026.s005.pdf]
