# Supplementary material for: Epstein–Barr virus-based plasmid enables inheritable transgene expression in mouse cerebral cortex
Source: PLoS One. 2021 Sep 30;16(9):e0258026. doi: 10.1371/journal.pone.0258026 (PMC8483300; doi:10.1371/journal.pone.0258026)
Supplement: Attachment — Submitted filename: Response to Reviewers.docx [file pone.0258026.s006.docx]

**Responses to Academic Editor:**

1) *Both reviewers and I, we very much like the study. However, the n-number in the most important experiments is too low. Both reviewers open different options to solve this major point by either increasing the n-number to four animals for statistical testing of the most important data sets (see the comments of both reviewers). Other data that are less relevant regarding the properties of the plasmids, meaning the data that are more descriptive, might be presented as single animal data (e.g. by showing representative images or countings for both animals). The focus should be put on the properties of the plasmids, as this is the main topic of the paper.*

First, in accordance with the academic editor’s and reviewers’ comments, quantifications and statistical tests as shown in Figures 1, 4, and 7 were redone after including additional data from one to three animals. The corresponding results did not affect the conclusions drawn. Accordingly, the numerical values in the text, figures, and figure captions have been modified. The details of each modification are as follows.

- Figure 1: Additional data from three mice for pCAG-GFP-EB-oriP transfection and two mice for pCAG-GFP (control) transfection were added for quantifications, and then statistical tests were redone. In total, five mice were investigated for pCAG-GFP-EB-oriP and four mice for pCAG-GFP.
- Figure 4: Additional data from one mouse for each of pCAG-GFP-EB-oriP and pCAG-GFP were added for quantifications, and then statistical tests were redone. In total, three mice were investigated for both pCAG-GFP-EB-oriP and pCAG-GFP.
- Figure 7: At P7, additional data from one mouse for each of shTBCD#1 and NS (non-silencing) were added for quantifications. Furthermore, at P14, additional data from one mouse for shTBCD#1, and two mice for each of shTBCD#2 and NS were added for quantifications. In total, three mice were investigated for both shTBCD#1 and NS at P7. Similarly, three mice were investigated for shTBCD#1, and four mice for each of shTBCD#2 and NS at P14. The statistical tests were redone based on these revised quantifications.

From the revised statistical test in Figure 7, the proportion of GFP-labeled superficial neurons in shTBCD#2 at P7 was significantly higher than in NS. Thus, the two sentences related to GFP-labeled superficial neurons in shTBCD#2 have been changed as follows (shown in red).

(page 26, lines 491–496)

**“~~The superficial neurons accounted for 45% of GFP-labeled cells, which was comparable to that of NS-transfected brains (39.7%).~~** Notably, as at P14, GFP fluorescence of superficial neurons was visibly weaker, coinciding with the faint GFP fluorescence of the brain. The proportion of GFP-labeled astrocytes was significantly lower (25.0% in NS, 13.6% in shTBCD#2), which consequently slightly raised the proportions of GFP-labeled **superficial neurons (39.7% in NS, 45.0% in shTBCD#2) and** cells in the V-SVZ (35.1% in NS, 37.4% in shTBCD#2).**”**

With regard to Figure 5, additional data from one mouse for separate transfection were added. In accordance with Reviewer #1’s suggestion, images of whole brains and sections have been shown per animal in the revised manuscript (see Figure 5B–F). At the same time, quantifications have been shown per animal in the graph, and then the error bars and symbols of statistical significance have been removed (see Figure 5G and its caption).

2) *Furthermore, please add more details about the post-surgery treatment (medication, analgesic treatment) of the experimental animals.*

The following sentences describing the details about the pre- and post-surgery treatment including anesthetic and analgesic medication for the experimental animals have been included in the section titled “In utero electroporation” in the materials and methods of the manuscript.

(page 35, lines 664–681)

**“****For *in utero* electroporation, ICR and C57BL6J mice were purchased from Japan SLC (Hamamatsu, Japan). Two to four pregnant mice were housed together for at least 2 days before surgery. The mice were anesthetized by the intraperitoneal injection of a mixture of three different anesthetic and analgesic agents (0.15 mg/kg medetomidine hydrochloride, 2.0 mg/kg midazolam, and 2.5 mg/kg butorphanol tartrate in 0.9% NaCl).** To prevent uterine muscle contraction during manipulation, 50–80 µl of 0.1 mg/ml ritodrine hydrochloride in 0.9% NaCl was evenly injected into the abdominal cavity. Next, 2–3 µl of plasmid DNA solution in HBS (20 mM Hepes, 150 mM NaCl, pH 7.4) (2–4 mg/ml with 0.01% Fast Green) was injected into the lateral ventricle of the embryo using a mouth-controlled micropipette, and electrical pulses were delivered using a NEPA21 electroporator (voltage: 30–33 V; pulse length: 30 ms; pulse intervals: 970 ms; repeated pulses: 5) with a tweezer-type electrode (CUY650P5; NepaGene, Ichikawa, Japan). **After surgery, the mice were warmed on a plate maintained at 38.5 °C with careful monitoring until they woke from the anesthesia. Then, the mice receiving surgery were returned to their original cage and housed together with nesting materials to prevent a decrease of body temperature. Two days before the expected date, each mouse was transferred to a new cage with part of the nest and nesting materials and housed individually.”**

**Responses to Reviewer #1:**

1) *Quantifications shown in figures 1, 4, 5 and 7 were performed only in 2 animals. This is very low, considering the high variability of experiments using in utero electroporations (IUE). Moreover, this sample size does not allow statistical comparisons.*

*I understand the amount of work involved in these experiments and don't expect that the authors increase the sample size for all those 4 different quantifications. However, the authors must definitely increase the sample sizes if they want to perform statistical comparisons. Otherwise, they must describe the data per animal.*

*In my opinion, an interesting approach could be:*

*Figure 1 and 4: increase samples sizes and perform statistical tests, since data described in these figures are important to validate the benefit of using the EB-oriP plasmid.*

*Figure 5: show results per animals. As I will detail below, the data described here is interesting, but can have different interpretations.*

*Figure 7: increase sample size in one time-point (P7 or P14) and perform statistical test.*

Thank you for this comment. First, in accordance with your suggestion, quantifications and statistical tests as shown in Figures 1, 4, and 7 were redone after including additional data from one to three animals. The corresponding results did not affect the conclusions drawn. Accordingly, the numerical values in the text, figures, and figure captions have been modified. The details of each modification are as follows.

- Figure 1: Additional data from three mice for pCAG-GFP-EB-oriP transfection and two mice for pCAG-GFP (control) transfection were added for quantifications, and then statistical tests were redone. In total, five mice were investigated for pCAG-GFP-EB-oriP and four mice for pCAG-GFP.
- Figure 4: Additional data from one mouse for each of pCAG-GFP-EB-oriP and pCAG-GFP were added for quantifications, and then the statistical test was redone. In total, three mice were investigated for both pCAG-GFP-EB-oriP and pCAG-GFP.
- Figure 7: At P7, additional data from one mouse for each of shTBCD#1 and NS (non-silencing) were added for quantifications. Furthermore, at P14, additional data from one mouse for shTBCD#1, and two mice for each of shTBCD#2 and NS were added for quantifications. In total, three mice were investigated for both shTBCD#1 and NS at P7. Similarly, three mice were investigated for shTBCD#1, and four mice for each of shTBCD#2 and NS at P14. The statistical tests were redone based on these revised quantifications.

The revised statistical test as shown in Figure 7 indicated that the proportion of GFP-labeled superficial neurons in shTBCD#2 at P7 was significantly higher than that of NS. Thus, the two sentences related to GFP-labeled superficial neurons in shTBCD#2 have been changed as follows.

(page 26, lines 491–496)

**“~~The superficial neurons accounted for 45% of GFP-labeled cells, which was comparable to that of NS-transfected brains (39.7%).~~** Notably, as at P14, GFP fluorescence of superficial neurons was visibly weaker, coinciding with the faint GFP fluorescence of the brain. The proportion of GFP-labeled astrocytes was significantly lower (25.0% in NS, 13.6% in shTBCD#2), which consequently slightly raised the proportions of GFP-labeled **superficial neurons (39.7% in NS, 45.0% in shTBCD#2) and** cells in the V-SVZ (35.1% in NS, 37.4% in shTBCD#2).**”**

With regard to Figure 5, additional data from one mouse for separate transfection was added. In accordance with Reviewer #1’s suggestion, images of whole brains and sections have been shown per animal in the revised manuscript (see Figure 5B–F). At the same time, quantifications have been shown per animal in the graph, and then the error bars and symbols of statistical significance have been removed (see Figure 5G and its caption).

2) *Electroporation of CreERT2 plasmids in reporter mouse lines has been largely used to stably label neocortical progenitors and their progeny through Cre-lox recombination. This can be combined with cell-type specific promoters and shRNAs, as described in the current manuscript for the EB-oriP. The authors should discuss the pros and contras of this latter method in comparison with Cre-lox based strategies.*

Thank you for pointing out another advantage of the EB-oriP plasmid. The possible applications of the EB-oriP plasmid in combination with Cre-loxP system have been described in the discussion of the revised manuscript as follows.

(page 33, lines 626–636)

**“IUE of the EB-oriP plasmid can be an additional experimental manipulation tool for clonal analysis and lineage tracing in combination with Cre-dependent multicolor fluorescent reporter mice [47]. The CreER^T2^ expression using the EB-oriP plasmid introduced by IUE can enable multicolor fluorescent labeling in the progeny cells of transfected progenitor cells at the time of tamoxifen administration. Furthermore, along with conditional fluorescent labeling with CreER^T2^, co-transfection with another EB-oriP plasmid coding a Cre-dependent transgene expression system, such as Cre-dependent excision of the STOP sequence shown in this study, can enable shRNA expression and genome modification by CRISPR/Cas9, as well as simple forced expression. In addition, as mentioned above, cell type-selective manipulation can be achieved by using cell type-specific promoters.”**

3) *Results described in Figure 7 are interesting, but highly preliminary. To support the interpretation that neurons transfected with shRNA against TBCD have a reduced survival, authors must provide more evidence, such as similar numbers of transfected neurons at earlier time-points (P0), increased proportions of dying neurons in the first postnatal week, etc.*

Your point is perfectly reasonable. However, the suggested experiments were not performed since looking in more detail at TBCD knockdown experiments was outside the scope of this study. Meanwhile, the other possibilities including progressive reduction of GFP expression and off-target effects in transfected cells have not been ruled out at all. Therefore, the following sentences shown in red have been added in the discussion of the revised manuscript.

(page 33, lines 613–619)

“These observations may help not only to understand the role of TBCD in cerebral cortex development, but also to explain the mechanism of impaired brain development caused by TBCD mutation in humans. **Meanwhile, the experimental results in this study are insufficient to draw definitive conclusions on the physiological function of TBCD. Further detailed investigations are necessary to understand the role of TBCD in brain development and the pathogenic effect of TBCD mutations in human patients.**”

4) *The discussion about progenitor heterogeneity needs to be tuned down. An alternative explanation for the observations described in Figure 5 could be that only a small fraction of NPCs transfected at E13 would remain as NPCs at E15 and generate upper layer neurons and astrocytes, whereas the vast majority of E13 progenitors would generate deep layer neurons and be depleted. Therefore, the low frequency of co-transfceted cells in the "separate electroporation" experiment could be explained by the low number of electroporated cells analysed. It is important to note here that every 1000 cells counted at P21 could indicate the progeny of less than 100 NPCs electroporated at E13/15. Moreover, as pointed out above, these quantifications were made only in 2 animals.*

Thank you for highlighting this critical issue. As you mentioned, the results shown in Figure 5 have another possible explanation. Therefore, in accordance with your comment, the sentences related to progenitor heterogeneity in the discussion have been revised as follows (shown in red).

(page 34, lines 638–644)

“Heterogeneity of NPCs could be ~~may be~~ reflected in electroporation susceptibility

The proportion of astrocytes from NPCs transfected with the EB-oriP plasmid using IUE at E13 was significantly lower than that at E15 (Fig. 4). Furthermore, the cells in the V-SVZ transfected at E13 appear to have become ~~became~~ less susceptible to IUE at E15 (Fig 5). These results support the notion ~~suggested~~ that the NPC populations susceptible to IUE at E13 and E15 have different properties regarding progeny production.”

**Responses to Reviewer #2**

1) *_ for most experiments, the number of biological replicates is 2 (Figures 1, 4, 5, 7) and this appears a low number. As many timepoints have been analyzed, it would be important to add at least one more biological replicate to the most important experiments, e.g., Figure 1F, Figure 4C.*

Thank you for pointing this out. First, in accordance with your suggestion, quantifications and statistical tests as shown in Figures 1, 4, and 7 were redone after including additional data from one to three animals. The corresponding results did not affect the conclusions drawn. Accordingly, the numerical values in the text, figures, and figure captions have been modified. The details of each modification are as follows.

- Figure 1: Additional data from three mice for pCAG-GFP-EB-oriP transfection and two mice for pCAG-GFP (control) transfection were added for quantifications, and then statistical tests were redone. In total, five mice for pCAG-GFP-EB-oriP and four mice for pCAG-GFP were investigated.
- Figure 4: Additional data from one mouse for each of pCAG-GFP-EB-oriP and pCAG-GFP were added for quantifications, and then the statistical test was redone. In total, three mice were investigated for both pCAG-GFP-EB-oriP and pCAG-GFP.
- Figure 7: At P7, additional data from one mouse for each of shTBCD#1 and NS (non-silencing) were added for quantifications. Furthermore, at P14, additional data from one mouse for shTBCD#1, and two mice for each of shTBCD#2 and NS were added for quantifications. In total, three mice for both shTBCD#1 and NS at P7 were investigated. Similarly, three mice were investigated for shTBCD#1, and four mice for each of shTBCD#2 and NS at P14. The statistical tests were redone based on these revised quantifications.

The revised statistical test as shown in Figure 7 indicated that the proportion of GFP-labeled superficial neurons in shTBCD#2 at P7 was significantly higher than that of NS. Thus, the two sentences related to GFP-labeled superficial neurons in shTBCD#2 have been changed as follows (shown in red).

(page 26, lines 491–496)

**“~~The superficial neurons accounted for 45% of GFP-labeled cells, which was comparable to that of NS-transfected brains (39.7%).~~** Notably, as at P14, GFP fluorescence of superficial neurons was visibly weaker, coinciding with the faint GFP fluorescence of the brain. The proportion of GFP-labeled astrocytes was significantly lower (25.0% in NS, 13.6% in shTBCD#2), which consequently slightly raised the proportions of GFP-labeled **superficial neurons (39.7% in NS, 45.0% in shTBCD#2) and** cells in the V-SVZ (35.1% in NS, 37.4% in shTBCD#2).**”**

With regard to Figure 5, additional data from one mouse for separate transfection was added. In accordance with the suggestion from the editor and another reviewer, images of whole brains and sections have been shown per animal in the revised manuscript (see Figure 5B–F). At the same time, quantifications have been shown per animal in the graphs, and then the error bars and symbols of statistical significance have been removed (see Figure 5G and its caption).

2) *_ along this line, a section describing the statistical analysis (e.g., how many sections per animal?) would be helpful.*

For quantification and statistical analysis, GFP- and/or RFP-labeled cells in two to five sections from the central part of the transfected region per animal were counted. The details have been described in each figure caption (see captions of Figures 1, 4, 5, and 7) as follows.

- Figure 1F: Two to four sections from the central part of the transfected region per animal were counted.
- Figure 4C: Two to three sections from the central part of the transfected region per animal were counted.
- Figure 5G: Two to five sections from the central part of the transfected region per animal were counted.
- Figure 7C: Two to three sections from the central part of the transfected region per animal were counted.

3) *_ In the use of cell-specific promoters (mGFAP, Figure 6), some GFP+ cells (electroporated) are also RFP (supposedly, GFAP+): can the author quantify the proportion of RFP+/GFP+ over time (P5, P14 and P21)? This because it’s known that in the mouse cortical gray matter GFAP expression declines with mice becoming adult. This would indeed further support the use of this system as tool to investigate physiological properties of brain cells.*

In accordance with your suggestion, the number of GFP-labeled cells that were co-labeled with RFP was counted and subjected to attempted quantification at P5, P14, and P21 from three mice at each time. However, as described below in detail, the number of GFP-labeled cells in cortical gray matter of observed sections, namely, GFP-labeled astrocytes, was insufficient for quantification. Therefore, mentioning the number of co-labeled cells, especially in relation to the developmental change of GFAP expression, is thought to be impossible and inappropriate. Thus, no change has been made in regard to this comment. Regarding the low number of GFP-labeled astrocytes, as described in the manuscript, GFP expression as shown in Figure 6 does not occur unless both Acceptor and Donor plasmids are co-transfected. Thus, the low number of GFP-labeled cells is thought to be due to the lower efficiency of co-transfection.

The counts of GFP-labeled cells and co-labeled cells

- In the gray matter, 63 of 140 cells at P5, 29 of 90 cells at P14, and 30 of 96 cells at P21 of GFP-labeled astrocytes were co-labeled with RFP.
- In the V-SVZ, 70 of 164 cells at P5, 51 of 73 cells at P14, and 19 of 41 cells at P21 of GFP-labeled cells were co-labeled with RFP.
- None of the GFP-labeled superficial neurons (1170 cells at P5, 653 cells at P14, and 666 cells at P21) were co-labeled with RFP at all ages.

4) *_ in the TBCD knock-down experiment (Figure 7), a progressive reduction of GFP expression is observed. Can the author stain for EBNA1 (like in figure S1E) to ascertain whether the loss of signal is not due to a reduction in GFP expression, but a loss of cells? If that’s the case, can the author analyze cell death between P1 and P7 (e.g., P3 or P4)? This would reinforce the hypothesis that TBCD knock-down causes the loss of cells.*

Your point is perfectly reasonable. However, the suggested experiments were not performed since looking in more detail at TBCD knockdown experiments was outside the scope of this study. Meanwhile, the other possibilities including progressive reduction of GFP expression and off-target effects in transfected cells have not been ruled out at all. Therefore, the following sentences shown in red have been added in the discussion of the revised manuscript.

(page 33, lines 613–619)

“These observations may help not only to understand the role of TBCD in cerebral cortex development, but also to explain the mechanism of impaired brain development caused by TBCD mutation in humans. **Meanwhile, the experimental results in this study are insufficient to draw definitive conclusions on the physiological function of TBCD. Further detailed investigations are necessary to understand the role of TBCD in brain development and the pathogenic effect of TBCD mutations in human patients.**”

5) *_ this system seems particularly suitable for clonal analysis: has the author tried to electroporate ng (instead of microgram) of plasmidic DNA, and assess whether clonality can be observed (e.g., 1 or very few progenitor cells giving rise to intermediate progenitors, neurons, and astrocytes)? This could be a potential application worth discussing.*

Thank you for pointing out another advantage of the EB-oriP plasmid. I have not attempted to electroporate the EB-oriP plasmid at a low dose (ng or less). The possible applications of the EB-oriP plasmid for clonal analysis, particularly in combination with the Cre-loxP system, have been described in the discussion of the revised manuscript as follows.

(page 33, lines 626–636)

**“IUE of the EB-oriP plasmid can be an additional experimental manipulation tool for clonal analysis and lineage tracing in combination with Cre-dependent multicolor fluorescent reporter mice [47]. The CreER^T2^ expression using the EB-oriP plasmid introduced by IUE can enable multicolor fluorescent labeling in the progeny cells of transfected progenitor cells at the time of tamoxifen administration. Furthermore, along with conditional fluorescent labeling with CreER^T2^, co-transfection with another EB-oriP plasmid coding a Cre-dependent transgene expression system, such as Cre-dependent excision of the STOP sequence shown in this study, can enable shRNA expression and genome modification by CRISPR/Cas9, as well as simple forced expression. In addition, as mentioned above, cell type-selective manipulation can be achieved by using cell type-specific promoters.”**

*Minor point:*

1) *_ can the author explain in the figure legends when pseudo-color is used? For instance, in Figure 2E, the big panel shows green (GFP) and violet (Dcx): however, in the RMS violet and white color are shown. Likewise, in Figure 2F, Figure 4E and 4F.*

In Figures 2D, E, F, and 4E and F, violet is used to show the RMS and olfactory bulb as DCX-immunopositive parts. In Figure 2F, white is used to sharply show the morphologies of individual GFP-expressing cells in the RMS and OB. To show the area (RMS and OB) where the GFP-expressing cells were observed, GFP images were colored green (GFP) and merged with DCX images colored violet.
